# Pulse Detecting Genetic Circuit – A New Design Approach

**DOI:** 10.1371/journal.pone.0167162

**Published:** 2016-12-01

**Authors:** Nasimul Noman, Mara Inniss, Hitoshi Iba, Jeffrey C. Way

**Affiliations:** 1 School of Electrical Engineering and Computer Science, The University of Newcastle, Callaghan, NSW, Australia; 2 Department of Systems Biology, Harvard Medical School, Boston, MA, United States of America; 3 Department of Information and Communication Engineering, Graduate School of Information Science and Technology, The University of Tokyo, Bunkyo, Tokyo, Japan; 4 Wyss Institute for Biologically Inspired Engineering, Boston, MA, United States of America; Niels Bohr Institute, DENMARK

## Abstract

A robust cellular counter could enable synthetic biologists to design complex circuits with diverse behaviors. The existing synthetic-biological counters, responsive to the beginning of the pulse, are sensitive to the pulse duration. Here we present a pulse detecting circuit that responds only at the falling edge of a pulse–analogous to negative edge triggered electric circuits. As biological events do not follow precise timing, use of such a pulse detector would enable the design of robust asynchronous counters which can count the completion of events. This transcription-based pulse detecting circuit depends on the interaction of two co-expressed lambdoid phage-derived proteins: the first is unstable and inhibits the regulatory activity of the second, stable protein. At the end of the pulse the unstable inhibitor protein disappears from the cell and the second protein triggers the recording of the event completion. Using stochastic simulation we showed that the proposed design can detect the completion of the pulse irrespective to the pulse duration. In our simulation we also showed that fusing the pulse detector with a phage lambda memory element we can construct a counter which can be extended to count larger numbers. The proposed design principle is a new control mechanism for synthetic biology which can be integrated in different circuits for identifying the completion of an event.

## Introduction

Synthetic biology borrows the basic principles from engineering and molecular biology, and applies these principles in designing, testing, validating and assembling genetic parts into larger systems [1]. Over the past 15 years synthetic biology researchers have designed numerous synthetic genetic circuits and a trend of increasing circuit complexity seems likely [2]. The design principles of electrical circuits have inspired and have been incorporated in the construction of many synthetic genetic circuits [3,4, 5, 6]. Like in electrical circuits, memory is an essential functional unit in biological systems which records the received stimulus and directs the cell fate in alternate directions based on the logged experience. Consequently, a diverse design approach has been exercised in registering a biological event in a cell and probing the record at a later time [4, 7, 8].

A counter is another basic device that track events and is extensively used in building a wide range of complex electrical circuits. The existence of counting mechanism in wild organisms has been documented [9, 10]. With the help of a robust cellular counter, synthetic biologists could design novel control mechanisms and applications based on the occurrence of events. A few successful circuits have been constructed [11, 12]. The design of a counter makes the use of memory and a single memory unit can work as a counter capable of counting a single event. Such a one-counter can be cascaded to count numbers larger than one but counting high numbers will be challenging because the number of orthogonal systems will increase linearly with the maximum number we want to count. One way to overcome this difficulty is to use set-reset memory devices as shown by Subsoontorn and Endy [12].

Another potential challenge in designing a robust biological counter is the ability to count at completion of the event. The existing designs of the counters are sensitive to the pulse duration–a brief pulse will be ignored and a lengthy pulse can cause the counter to count ahead [11]. This problem can be evaded if we can design a counter that advances the count at the end of the pulse, as is the common practice in electrical counter design [13]. The essential component in such a design is a pulse detecting circuit that responses only at the falling edge of the pulse stimulus. Use of such a pulse detector will make the counter robust to pulse duration.

In this work, we present a design of the robust genetic pulse detector using the lambda CI repressor protein [14]. By preventing the dimerization of CI protein until the triggering pulse completes, we identify the end of the event and subsequently the dimerized CI protein will trigger the reporter circuit. In simulation we tested and characterized the pulse detecting device to identify the limit of its operation. We designed an extendable one-counter by coupling this pulse detecting circuit with a lambda switch based memory [7]. Using a detailed chemical modeling and stochastic simulation we show that the presented robust pulse detector works with practical biologically parameters and can be used in designing falling edge triggered genetic counter.

## A new design control for pulse detection

In principle, it is possible to design an asynchronous counter using both negative edge triggering (NET) and positive edge triggering (PET). However, in electronics most of the asynchronous counters are designed using NET because it makes the linking to flip-flops easier which should change state when the previous bit changes from high to low. An additional advantage of designing counters with NET is that they count events irrespective of the event’s duration and frequency. The design of counters presented by Friedland et al. [11] is analogous to synchronous counters found in digital systems, and counted correctly only in response to pulses of defined duration. In contrast, the design of the counter outlined in [12] corresponds to asynchronous counters. A pulse detector circuit that triggers only at the falling edge of a pulse would facilitate the design of an asynchronous counter and can be used in designing many other genetic circuits.

We designed a pulse detector circuit that uses distinctive characteristics of the lambda CI repressor protein to explore design considerations for a transcription-based biological negative edge detector. The bacteriophage lambda has a complex set of interlocking regulatory mechanisms that it uses to maintain the lysogenic state and to transition to the lytic state [14, 15]. In the lysogenic state the lambda genome is integrated in the chromosome of host cell and replicated with cell division. In response to a DNA-damage signal, the lambda-phage exits the stable lysogenic state and enters the lytic state in which the phage lyses the cell, producing many new phage particles [14]. One regulatory module in lambda genome, colloquially known as lambda switch, mediates this decision and consists of: *cI* and *cro* genes, two promoters (P_RM_ and P_R_ transcribing *cI* and *cro* respectively) and three operators (O_R1_, O_R2_, and O_R3_) in the O_R_ region [15]; the three operators in the O_R_ region enhance the cooperativity of the system with respect to *cI* and allow a hair-trigger response in switching from the CI-rich state to the Cro-rich state [16].

Both CI and Cro proteins bind to the three operators with different affinities and control the transcription of *cI* and *cro* genes. RNA polymerase can transcribe gene *cro* when both O_R1_ and O_R2_ are free; similarly gene *cI* is transcribed when O_R3_ is free. CI protein can enhance the transcription from P_RM_ promoter when bound to O_R2_. A moderate level of CI protein is maintained by shutting down the P_RM_ promoter when CI level crosses a certain threshold. The double negative feedback mechanism along with the positive feedback from CI controls the expression of only one of the two genes (*cI* and *cro*) repressing the other and thereby allows the lambda phage maintaining its lysogenic state and switching to the lytic state [17]. These features allowed Kotula et al. [8] to construct a memory element based on switching from the CI state to the Cro state. These authors also noted that the Cro state was quite stable, at least under the conditions tested. Thus, switching from the Cro to the CI state could also be used to record events; this is the approach used here.

One characteristic of CI and Cro proteins, important for our design, is that they bind to the O_R_ operator sites in their dimer and higher-order multimers only; monomers have no activity. Therefore activation and repression of these P_R_ and P_RM_ promoters could be controlled by preventing the dimer formation of CI and Cro proteins. This is a key element of the genetic device we present here. In their study on operator and non-operator DNA binding of lambda repressor protein CI, Nelson and Sauer isolated a mutant of CI repressor bearing a mutation in the DNA binding surface, Asn55Lys (N55K) that eliminated the binding affinity of the CI-mutant to operator sites but increased the affinity to non-specific DNA binding sites [18]. We recently demonstrated that CI (N55K) acts as a dominant negative inhibitor of the CI protein itself [19], presumably by forming mixed dimers as has been observed for the tet and lac repressors [20]. This mutation should not affect the dimerization characteristics of the protein. We refer to this protein as dominant-negative CI (CI_DN_) which is used for blocking dimer formation of CI proteins. Inhibition of the activity of a transcription factor by complexation with a dominant negative partner has previously been found useful [21]. Another protein that can be used to block the activity of lambda CI protein is the Antirepressor of P22, which appears to inactivate numerous lambdoid phage repressors [22].

**Fig 1:**
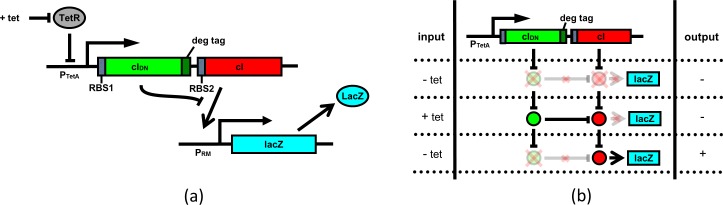
Schematic representation of the negative edge triggered pulse detecting circuit. (a) Components of the pulse detecting circuit. The central element is an artificial operon in which a regulated promoter directs transcription of high levels of an unstable inhibitor protein and lower levels of a target transcriptional regulator. In the specific version shown here, the tetracycline-regulated TetA promoter directs transcription of, firstly, a dominant-negative mutant of the lambda *cI* gene with a degradation tag (green), and secondly an intact version of the *cI* gene (red). TetR, the tetracyline repressor (gray), blocks transcription of this unit and P_RM_ (which is activated by intact CI protein) transcribing *lacZ* (sky-blue) serves as an illustrative readout of circuit activity. (b) Behavioral characteristics of the circuit in response to an inducing pulse, with time proceeding downward. In the initial absence of the inducer tetracycline, neither of the proteins is made and *lacZ* is OFF. Upon addition of tetracycline, both proteins are made and the CI_DN_ protein inhibits the wild-type protein, so *lacZ* remains OFF. Upon removal of the inducer, the CI_DN_ protein is rapidly degraded while the wild-type CI protein remains intact, and activates *lacZ* transcription.

The architecture of the pulse detecting circuit, assumed to be hosted in *E coli* bacterium, is shown in Fig 1(A). We placed both the wild-type *cI* and *cI*_*DN*_ under the control of a single inducible promoter. A degradation tag is added with *cI*_*DN*_ to ensure quick degradation of the monomeric proteins. One obvious candidate for the inducible promoter could be the *TetA* promoter P_TetA_ [23]. A much stronger RBS (RBS1) is required for *cI*_*DN*_ than the RBS (RBS2) for *cI*. Experimentally one would use a reporter such as the *lacZ* gene under the control of P_RM_ promoter. Essentially, the system works as follows: when the P_TetA_ is induced (during the pulse), CI_DN_ and CI transcripts are produced. Because of the stronger RBS associated with CI_DN_, many more CI_DN_ molecules are present in the cell compared to CI molecules. Therefore, almost all of the CI monomers will form heterodimers with CI_DN_, and there will be no CI_2_ to activate P_RM_ promoter. After the induction period, because of the degradation tag CI_DN_ molecules degrade quickly giving CI molecules a chance to form dimers and activate P_RM_ promoter. Fig 1(B) explains the input output relationship for the pulse detecting circuit.

In our stochastic simulation, we analyzed the pulse detecting circuit to determine the range of parameters (e.g. relative strength of the RBS sites, degradation tag efficiency) of the model for which the circuit produced the desired behavior. After successful model validation, we combined the pulse counter with a lambda phage memory element to construct a one-counter circuit. The simulation results show, when parameters such as RBS strengths are in the right range, that the designed circuit is able to count the event completion and can be expanded to count larger numbers.

## Results and Discussion

### Relative strength of RBS sites

**Fig 2:**
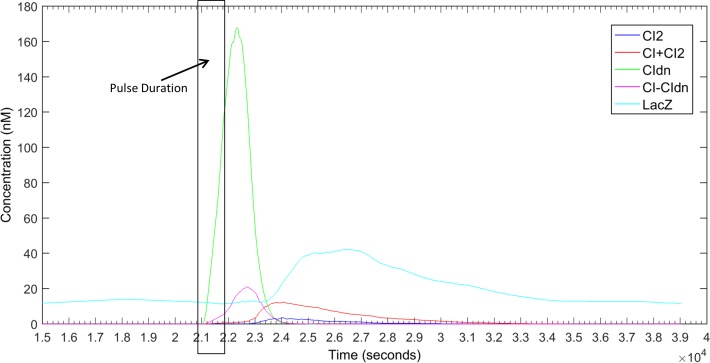
Response of the pulse detector circuit in Fig 1 for a pulse duration of ½ bacterial cell-cycle (CC) [1020 sec]. The pulse was activated at 10.2 CC (20808 sec) and deactivated at 10.7 CC (21828 sec). The relative strength of RBS1 and RBS2 was 20:1 and the degradation tag had half-life of 4 minutes. The response is average of 20 simulation runs.

In order to prevent CI proteins from activating the P_RM_ promoter, we need to block the homo-dimerization of wild type CI proteins. In our design, we plan to produce enough CI_DN_ proteins so that all CI wild-type proteins will form heterodimers with CI_DN_ rather homo-dimers. Since both the wild type and dominant type *cI* genes are transcribed from the same promoter P_TetA_ the best way to achieve that is to use RBS sites with different strength with *cI* and *cI*_*DN*_ genes. In our theoretical calculation it was found that the RBS of *cI*_*DN*_ (RBS1) should be at least 10 times stronger than the RBS of *cI* (RBS2). In order to verify that we tested our model with a range of RBS1:RBS2 strength ratios. It was found that if the strength ratio between RBS1 and RBS2 is 20:1 or greater, it is possible to prevent the dimer formation of CI proteins completely and thereby the reporter gene *lacZ* becomes activated only when the pulse is finished. Fig 2 shows the simulation of the pulse detector circuit with RBS1:RBS2 = 20:1. As the figure shows, the abundance of CI_DN_ molecules ensures that no CI_2_ dimer is formed to activate P_RM_ promoter. After the pulse, the degradation tag attached to *cI*_*DN*_ quickly removes CI_DN_ protein molecules from the cell allowing CI to form homo-dimers and trigger the reporter circuit. In our simulations, we varied the RBS1:RBS2 ratio from 2 to 25, and it was found that if it is less than 20 then the pulse detection might not work very precisely. As an example the results for the RBS1:RBS2 = 10 is included in S1 Fig. As we can see if RBS1:RBS2 is less than 20 then we have some CI_2_ in the system before the pulse is finished thus the reporter circuit might start to respond earlier. The effect is more visible for lengthier pulse durations as will be discussed later. In order to compare the effect of RBS strength ratios directly we put all the LacZ responses and the corresponding CI_2_ concentration changes in S2 Fig. From those response curves it is clear that if the RBS1:RBS2 ratio is less than 20 then CI_2_ concentration start to rise before the pulse finishes and reporter circuit starts to respond accordingly.

### Influence of degradation tag associated with *cI*_*DN*_

**Fig 3:**
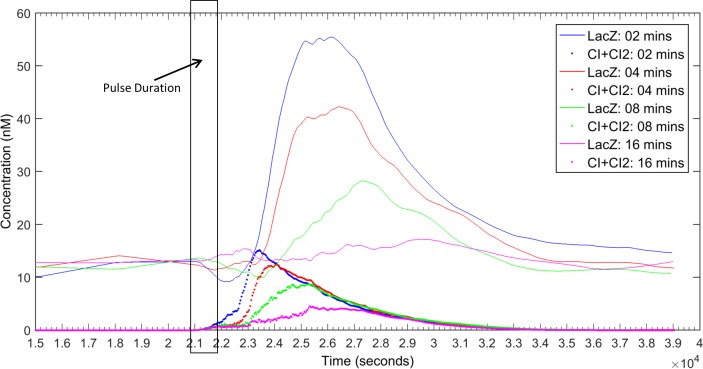
Response of the pulse detector circuit in Fig 1 with degradation tags of different half-lifes (2, 4, 8 and 16 minutes). For brevity only responses of LacZ and CI molecules were displayed for each deg-tag using the same color. The pulse was activated at 10.2 CC (20808 sec) and deactivated at 10.7 CC (21828 sec). The relative strength of RBS1 and RBS2 was 20:1. The response is average of 20 simulation runs.

The second most important challenge in the design is to quickly remove CI_DN_ from the cell after the completion of the pulse, so that we have enough CI concentration present in the cell to induce the reporter circuit. Evidently, adding a degradation tag to *cI*_*DN*_ is a workable solution. However, it should be noted that attaching a degradation-tag will also affect the concentration of CI_DN_ during the pulse. So we need a well-chosen degradation-tag so that we have sufficient CI_DN_ concentration to prevent formation of CI_2_ during the pulse and after the pulse the CI_DN_ molecules are quickly removed from the system. We therefore, experimented with various degradation-tags of different strengths. We run simulations with degradation-tags with half-life 2, 4, 8 and 16 minutes. The effect of the strength of degradation-tag is shown in Fig 3. From Fig 3, it is found that if degradation-tag is too strong (e.g. half-life 2 mins) then CI molecules start to form dimers before the pulse is finished and if the tag is too weak (e.g. half-life 16 mins) then CI_DN_ remains in the cell for long after the pulse and does not allow formation of CI-dimers to activate the reporter circuit. The degradation-tag with half-life of 4 minutes matches well with the RBS1:RBS2 = 20 to maintain CI_DN_ concentration high enough to prevent formation of CI_2_ during the pulse and quickly eradicate CI_DN_ from the cell to activate the reporter circuit after the pulse. S3 Fig shows that the combination of deg-tag of 4 minutes and RBS1:RBS2 ratio of 20 or higher is effective in building a working model for the pulse detecting circuit.

### Effect of pulse length

**Fig 4:**
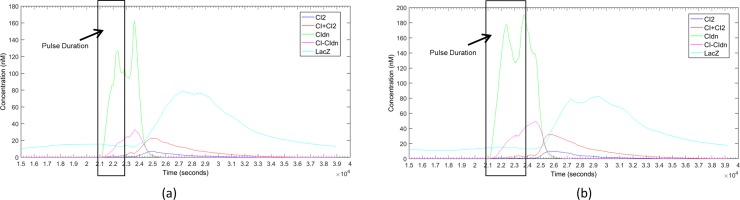
Response of the pulse detector circuit in Fig 1 for different pulse durations. The RBS1:RBS2 ratio was set to 20 and a deg-tag of 4 minutes was used. Each response in the graph is the average of 20 simulations. (a) The pulse was activated at 10.2 CC (20808 sec) and deactivated at 11.2 CC (22848 sec) [duration 1 bacterial cell-cycle (CC)]. (b) The pulse was activated at 10.2 CC (20808 sec) and deactivated at 11.7 CC (23868 sec) [duration 1 ½ bacterial cell-cycle (CC)].

Another important characteristic of the proposed pulse detector is its insensitivity to pulse duration. Since the circuit responses at the falling edge of the pulse it is not affected by the length of the pulse. In order to confirm that ability of the designed circuit we simulated the circuit with different pulse duration, specifically with pulses of 1.0 CC (cell-cycle) and 1.5 CC. Fig 4 shows the response of the designed pulse detector circuit with a RBS1:RBS2 ratio of 20 and 4 minutes degradation tag. Although the duration of the pulse was made double (Fig 4(A)) and triple (Fig 4(B)) there was no significant presence of CI_2_ in the cell to activate the *lacZ* reporter throughout the pulse. Consequently the *lacZ* responded only after the pulse was finished making the circuit independent of the pulse duration. We also extensively studied the effect of other ratios of RBS1:RBS2 and strength of degradation tag for these two pulse lengths and the summary of those results are presented in S4 and S5 Figs. The observation was analogous to what we found in case of the pulse duration of ½ cell-cycle–if the circuit is designed with a RBS1:RBS2 ratio of 20 or more and a degradation tag with 4 minutes half-life then it will behave as a perfect pulse detector circuit irrespective to pulse duration.

### Design of a counter circuit with the embedded pulse detector

**Fig 5:**
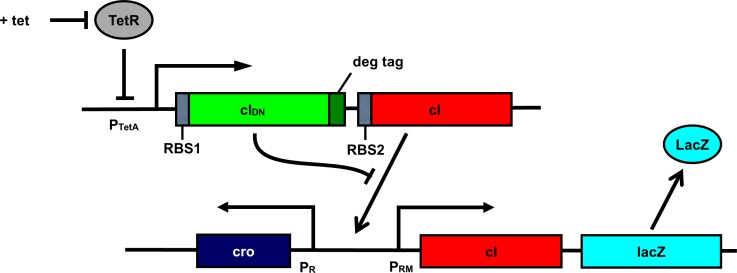
The design of the counter circuit. The two components of the design are the falling edge pulse detector circuit shown in Fig 1 and the lambda memory switch.

After we confirmed the reliability of the pulse detector circuit we fused a lambda memory circuit with it to design a synthetic counter. The bistable characteristic of lambda switch makes it a dependable memory device for recording the count after the pulse has been completed. The overall design of the counter circuit is shown in Fig 5. Initially the lambda memory is in Cro-rich state and retains that state until it is switched to CI-rich state in response to the completion of the pulse. With the beginning of the pulse which is simulated by the induction of the P_TetA_ promoter, mRNAs of both *cI*_*DN*_ and *cI* are transcribed. By the virtue of the stronger RBS1 enough CI_DN_ proteins are translated from *cI*_*DN*_ mRNA and those proteins form CI_DN_-CI dimers with CI monomers and prevent CI to form CI_2_ and activate the P_RM_ promoter. Now when the pulse finishes, the transcription of *cI*_*DN*_ and *cI* stops and the attached degradation-tag causes CI_DN_ to be removed quickly from the cell allowing CI molecules to form dimers. The CI-dimers interact with the P_RM_ promoter and switch the memory into CI-rich state from Cro-rich state. Once the memory has changed over to CI-rich state the feedback loops of lambda switch retains the memory in that state and thus records the completion of the pulse.

**Fig 6:**
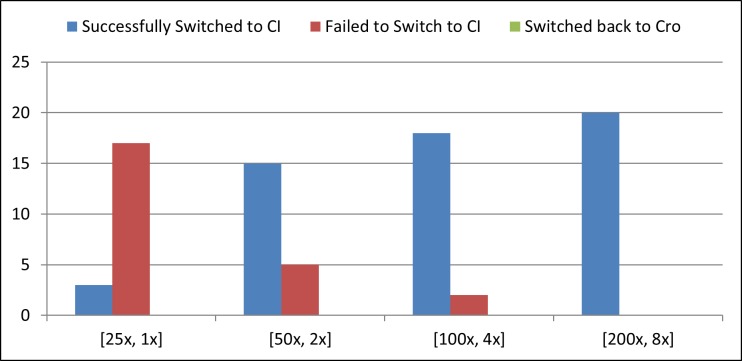
Success rate of switching the memory device ON. Each simulation was run 20 times with different [RBS1, RBS2] ratios: [25x,1x], [50x,2x], [100x,4x] and [200x,8x] where 25x means the RBS is 25 times stronger than the RBS in wild type *cI*.

**Fig 7:**
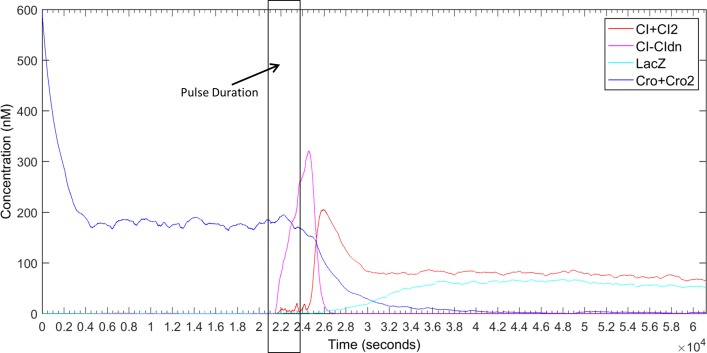
Average simulation of the 1 bit pulse counter circuit. The circuit was simulated for 30 bacterial CC (30X34 minutes). The duration of the pulse was 1.5 CC (1.5X34 minutes) and was activated at 10.2 CC (10.2X34 minutes).

However, for switching from Cro-rich state to CI-rich state we need sufficient amount of CI_2_ dimers present in the cell. According to some simulations we need approximately 70 ~ 100 nM CI for switching from Cro-rich state to CI-rich state. As shown in earlier sections, the strength ratio between RBS1 and RBS2 needed to be 20 or more to design a working pulse detector circuit. Taking that into consideration we experimented with different strengths of RBS1 and RBS2 so that we have a ratio of 25. We run our simulations under four conditions with [RBS1, RBS2] = [25x, 1x], [50x, 2x], [100x, 4x] and [200x, 8x] where 25x means that the RBS is 25 times stronger than the RBS of wild type *cI*. In every case, we used the degradation-tag with half-life of 4 minutes. Each simulation was run for 20 times and the summary of the results are shown in Fig 6. According to this simulation, if we have a 8 times stronger RBS attached to *cI* and a RBS 25 times stronger than that attached to *cI*_*DN*_ then we will be able to design a reliable counter circuit with the pulse detector circuit and the lambda memory circuit. Fig 7 shows the average simulation of the circuit with the following setting. Under this setting, in 20 out of 20 runs, the circuit successfully switched from Cro-rich state to CI-rich state. This also advocates the robustness of the counter circuit.

In simulation we have shown that it is possible to construct a counter circuit using the designed pulse detector circuit along with the lambda memory switch. However, the usage of lambda switch imposes additional requirements as the Cro-rich state of lambda is very stable and switching it to CI-rich state needs significant amount of CI present in the system. Therefore, we need to use very strong RBS both for *cI* and *cI*_*DN*_ genes so that we can get enough proteins per transcript. Alternatively, we can use a promoter with higher isomerization rate instead of P_TetA_. We can also consider stabilizing the *cI* and *cI*_*DN*_ mRNAs as well. It is also possible to change the OR regions in lambda switch that makes switching from Cro-rich to CI-rich state easier. Hence, the counter circuit can be constructed by using one of these strategies or applying their combinations altogether.

Furthermore, in order to show the robust behavior of the counter circuit in response to the pulse duration, we experimented with various pulse durations–in particular we varied the pulse from 1 CC, 2.5 CC, 5 CC and 10 CC where 1 CC means 1 bacterial cell cycle of 34 minutes. We repeated each simulation 20 times for each setup. The counter circuit exhibited very robust behavior by successfully counting each pulse irrespective to the pulse duration in each experimental run. The average simulation of this 1 bit pulse counter circuit for various pulse durations is shown in the S6 Fig. However, when the pulse duration was set 0.5 CC then in 7 runs out of 20 the circuit failed to switch to CI-rich state or switched back to Cro-rich state. These results indicates that if the pulse duration is very short then the current circuit cannot response, nevertheless, it is possible to design a counter circuit, based on the same principle, that can count shorter pulses by changing system parameters. The simulations in this section indicate that the designed counter circuit is capable of counting a pulse of any duration greater than a minimum limit.

**Fig 8:**
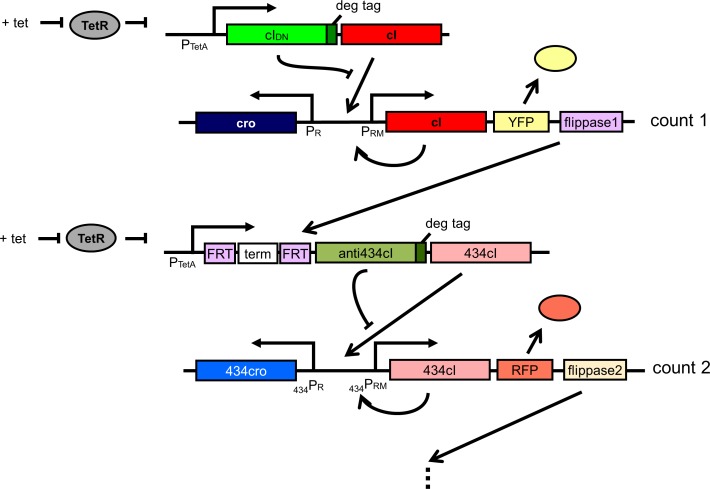
Design of two or higher bit counters using the pulse detector circuit.

The designed counter circuit with the aid of pulse detector circuit can be cascaded for building counters that can count larger numbers. Fig 8 shows a two-counting circuit that makes use of FLP-FRT recombination. The *flippase1* is transcribed from the P_RM_ promoter along with *YFP* and *cI* once the circuit has switched to CI-rich state (i.e. after it has counted one). The Flippase1 then can remove the terminator allowing the P_TetA_ promoter to transcribe CI and anti-CI proteins from 434. Subsequently the anti434CI and 434CI proteins can behave similar to CI and CI_DN_ and switch 434Cro state to 434CI state interacting with respective promoters. Another FLP-FRT pair (*flipppase2*) can be added for subsequent counting as shown in Fig 8. Moreover, the capability of the pulse detector circuit to respond to the falling edge of a pulse would enable us designing the asynchronous counter and many other useful synthetic circuits.

## Conclusion

This work presents a synthetic circuit for detecting the falling edge of a pulse based on the interaction between two proteins. The basic principle of this design is to co-express two proteins from an inducible promoter and one of the proteins will interfere with the activity of the other and prevent the second protein from its usual activity. The first protein will also have a degradation tag attached so that it will be quickly removed from the cell when the induction subdues and thus will allow the second protein to return to its natural action. In our design we used the lambda CI protein and one of its mutants which we call CI_DN_ for designing the pulse detector circuit. The interaction between CI and CI_DN_ has been characterized in laboratory experiments and it was found that the repression of CI by CI_DN_ occurs in a dose dependent manner [19]. Using stochastic simulation we showed that by selecting the RBS sites associated with *cI* and *cI*_*DN*_ and the degradation-tag attached to *cI*_*DN*_ we can construct a pulse detecting circuit that is robust to pulse duration. Since the biological events are not precisely timed, a pulse detecting circuit that can work irrespective to pulse duration would be very attractive to synthetic biologists. Fusing the pulse detector with lambda memory circuit we constructed a counter that can count the completion of an event. In our simulation the counter exhibited robust behavior. The design is generalized enough to be extended for constructing counter capable of counting higher numbers. Furthermore, the pulse detecting circuit can also be used for designing asynchronous biological counters.

The presented design is a new control mechanism for synthetic biology. The design principle can be used for many other circuits for detecting the completion of an event. Most of the verification of the design has been done in simulation but model based design has been used in previously designed synthetic circuits (e.g. toggle switch, repressilator). However, the results generated by the model could be successfully reproduced in experiments only after tuning the circuit. Although stochastic models represents the biological systems more closely compared to the deterministic models (e.g. differential equation based model), it is expected that some tuning of the model would be necessary to implement it in experiments [2]. Therefore, the designed pulse detecting circuit can be constructed *in vivo* perhaps with some necessary adjustments and can play as a valuable component for synthetic biology.

## Methods

We used a reaction based model for simulating different components of our circuits. Each model consists of a set of chemical reaction and we used Gillespie algorithm [24] for stochastic simulation of each model. The basic components of different models are: homo-dimerization of CI and Cro proteins and hetero-dimerization of CI-CI_DN_ proteins, binding of CI_2_ and Cro_2_ dimers to O_R_ operator sites (O_R1_, O_R2_ and O_R3_), binding of RNA polymerase to P_RM,_ P_R_ and P_TetA_ promoters, isomerization of different promoters, transcription of *cI* and *cI*_*DN*_, *cro* and *lacZ* from respective promoters and translation of those transcripts corresponding to associated RBS sites, degradation of mRNA molecules and protein monomers and dimers according to their half-life or attached degradation-tag’s half-life respectively.

All the model parameters are set using biochemical data. Most of the parameters came from the model of lambda switch by Morelli et al. [25]. Parameters for tet promoter came from [26]. The details of the model with chemical reactions and parameters are added in S1 File.

## Supporting Information

S1 Fig Response of the pulse detector circuit in Fig 1 for a pulse duration of ½ bacterial cell-cycle (CC) [1020 sec]. The pulse was activated at 10.2 CC (20808 sec) and deactivated at 10.7 CC (21828 sec). The relative strength of RBS1 and RBS2 was 10:1 and the degradation tag had half-life of 4 minutes. The response is average of 20 simulation runs.
(TIF)

S2 Fig Response of LacZ and CI molecules in the pulse detector circuit in Fig 1 for a pulse duration of ½ bacterial cell-cycle (CC) [1020 sec]. The pulse was activated at 10.2 CC (20808 sec) and deactivated at 10.7 CC (21828 sec). The relative strength of RBS1 and RBS2 was varied from 2 to 25. The half-life of the degradation tag was 4 minutes. The response is average of 20 simulation runs.
(TIF)

S3 Fig The effect of various degradation tags and RBS1:RBS2 ratio on the concentration of LacZ and CI molecules in the pulse detector circuit in Fig 1. The duration of the pulse, activated at 10.2 CC (20808 sec) and deactivated at 10.7 CC (21828 sec), is ½ bacterial cell-cycle. The relative strength of RBS1 and RBS2 was varied from 10, 15, 20 and 25. For each RBS1:RBS2 ratio degradation tags with half-life 2, 4, 8 and 16 minutes were used. The response is average of 20 simulation runs.
(TIF)

S4 Fig The effect of various degradation tags and RBS1:RBS2 ratio on the concentration of LacZ and CI molecules in the pulse detector circuit in Fig 1. The duration of the pulse, activated at 10.2 CC (20808 sec) and deactivated at 11.2 CC (22848 sec), is 1 bacterial cell-cycle. The relative strength of RBS1 and RBS2 was varied from 10, 15, 20 and 25. For each RBS1:RBS2 ratio degradation tags with half-life 2, 4, 8 and 16 minutes were used. The response is average of 20 simulation runs.
(TIF)

S5 Fig The effect of various degradation tags and RBS1:RBS2 ratio on the concentration of LacZ and CI molecules in the pulse detector circuit in Fig 1. The duration of the pulse, activated at 10.2 CC (20808 sec) and deactivated at 11.7 CC (23868 sec), is 1½ bacterial cell-cycle. The relative strength of RBS1 and RBS2 was varied from 10, 15, 20 and 25. For each RBS1:RBS2 ratio degradation tags with half-life 2, 4, 8 and 16 minutes were used. The response is average of 20 simulation runs.
(TIF)

S6 Fig The robust behavior of the 1 bit pulse counter circuit against the pulse duration. Each circuit was simulation for 30 bacterial CC (30X34 minutes).The duration of the pulse (each activated at 10.2 CC (20808 sec)) was varied from 1CC (2040 sec), 2.5 CC (5100 sec), 5 CC (10200 sec) and 10 CC (20400 sec). Each figure shows the average of 20 simulation runs.
(TIF)

S1 File Details of the counter circuit. (DOCX)
