# Supplementary figures and images for: Pulse Detecting Genetic Circuit – A New Design Approach
Source: PLoS One. 2016 Dec 1;11(12):e0167162. doi: 10.1371/journal.pone.0167162 (PMC5131961; doi:10.1371/journal.pone.0167162)

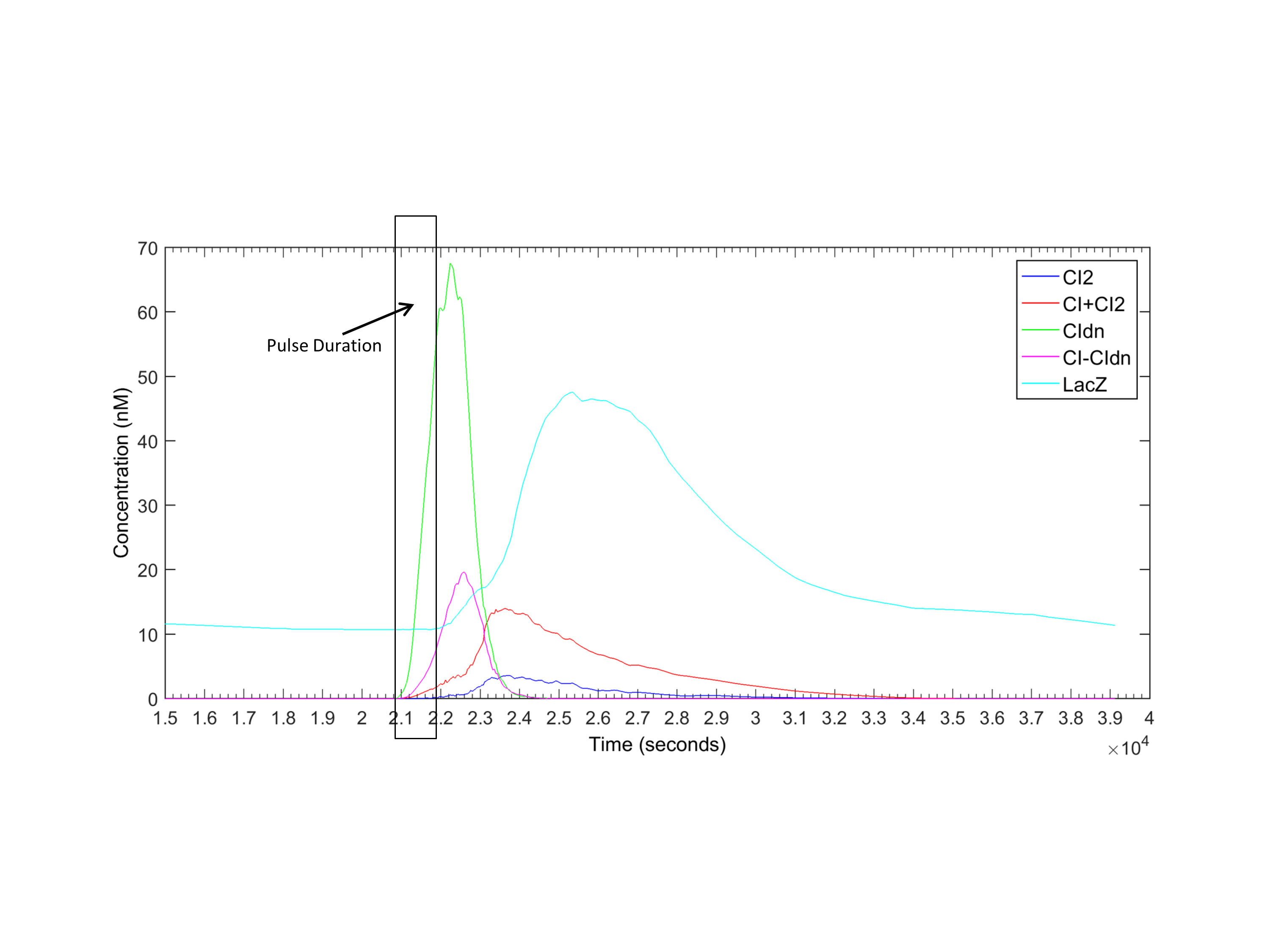

Supplement: S1 Fig — The pulse was activated at 10.2 CC (20808 sec) and deactivated at 10.7 CC (21828 sec). The relative strength of RBS1 and RBS2 was 10:1 and the degradation tag had half-life of 4 minutes. The response is average of 20 simulation runs. (TIF) [file pone.0167162.s001.tif]

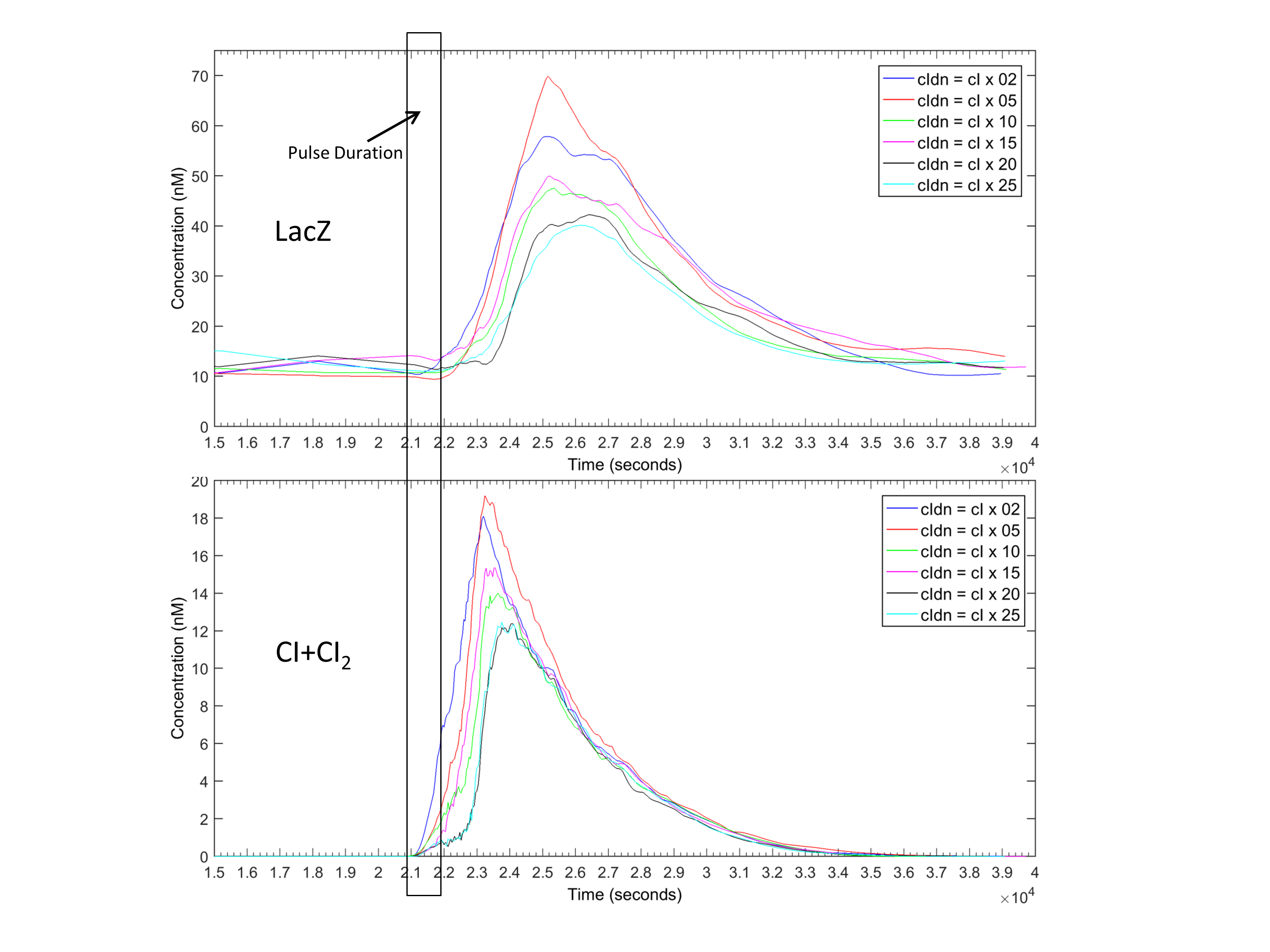

Supplement: S2 Fig — The pulse was activated at 10.2 CC (20808 sec) and deactivated at 10.7 CC (21828 sec). The relative strength of RBS1 and RBS2 was varied from 2 to 25. The half-life of the degradation tag was 4 minutes. The response is average of 20 simulation runs. (TIF) [file pone.0167162.s002.tif]

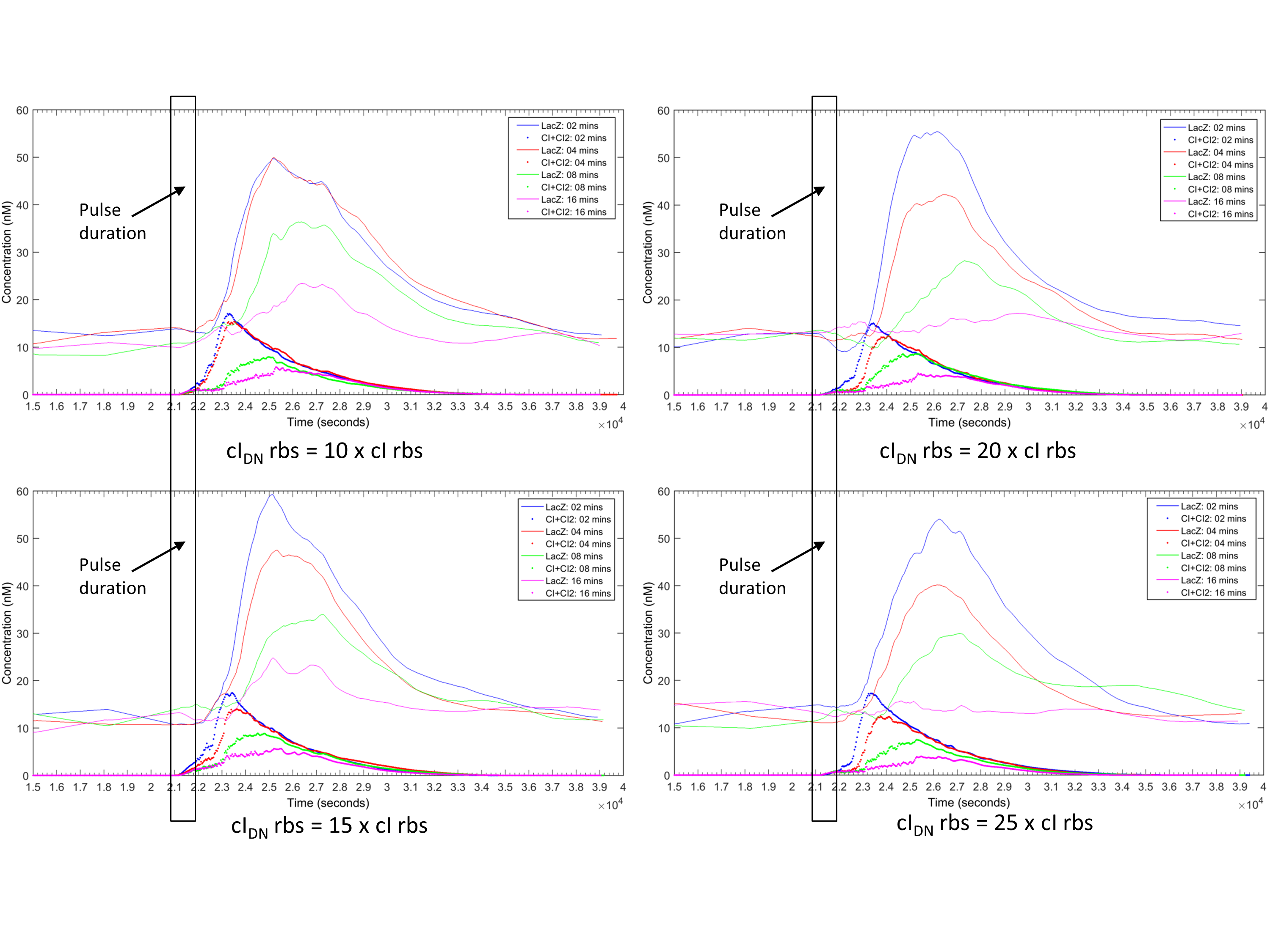

Supplement: S3 Fig — The duration of the pulse, activated at 10.2 CC (20808 sec) and deactivated at 10.7 CC (21828 sec), is ½ bacterial cell-cycle. The relative strength of RBS1 and RBS2 was varied from 10, 15, 20 and 25. For each RBS1:RBS2 ratio degradation tags with half-life 2, 4, 8 and 16 minutes were used. The response is average of 20 simulation runs. (TIF) [file pone.0167162.s003.tif]

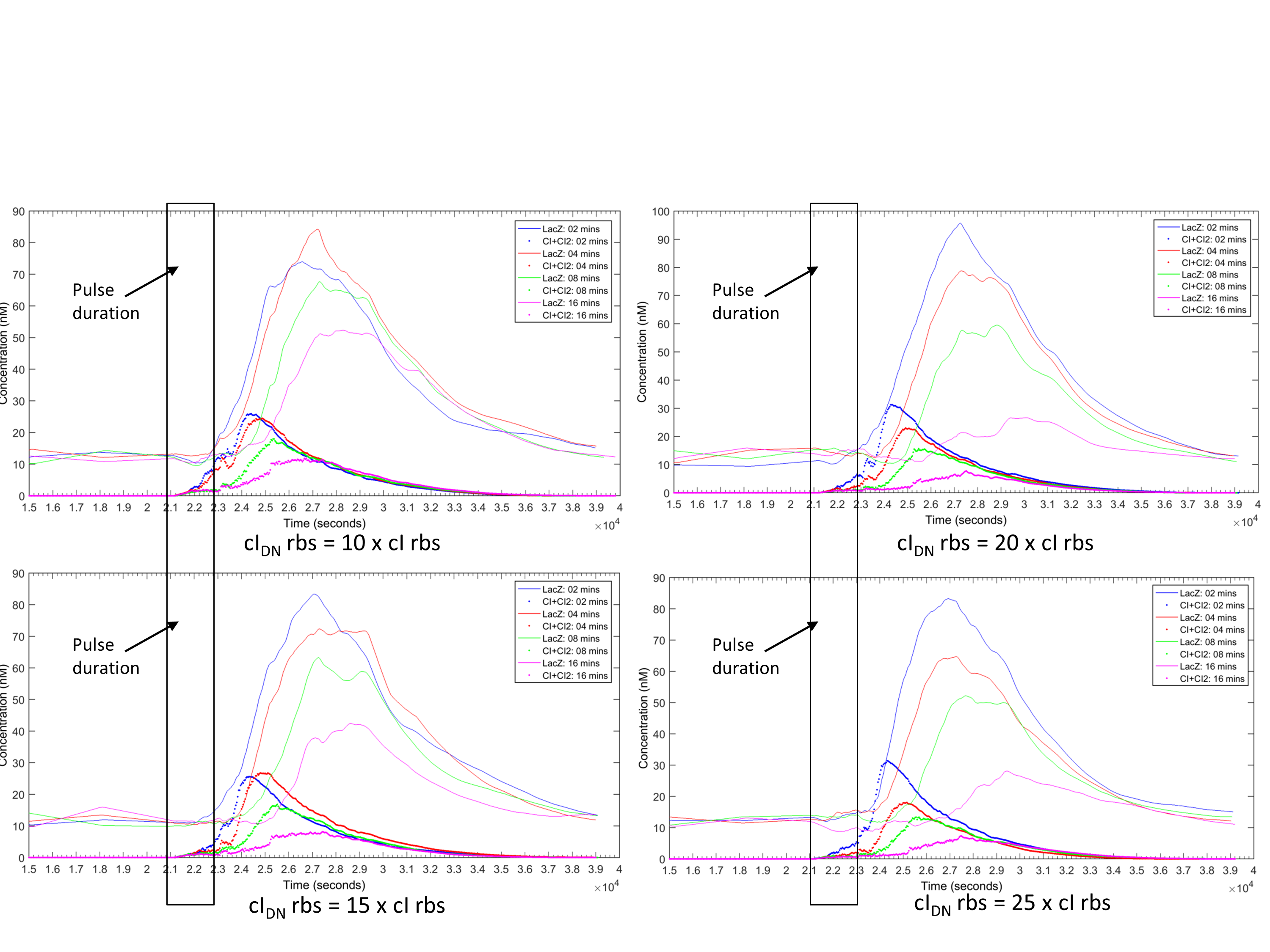

Supplement: S4 Fig — The duration of the pulse, activated at 10.2 CC (20808 sec) and deactivated at 11.2 CC (22848 sec), is 1 bacterial cell-cycle. The relative strength of RBS1 and RBS2 was varied from 10, 15, 20 and 25. For each RBS1:RBS2 ratio degradation tags with half-life 2, 4, 8 and 16 minutes were used. The response is average of 20 simulation runs. (TIF) [file pone.0167162.s004.tif]

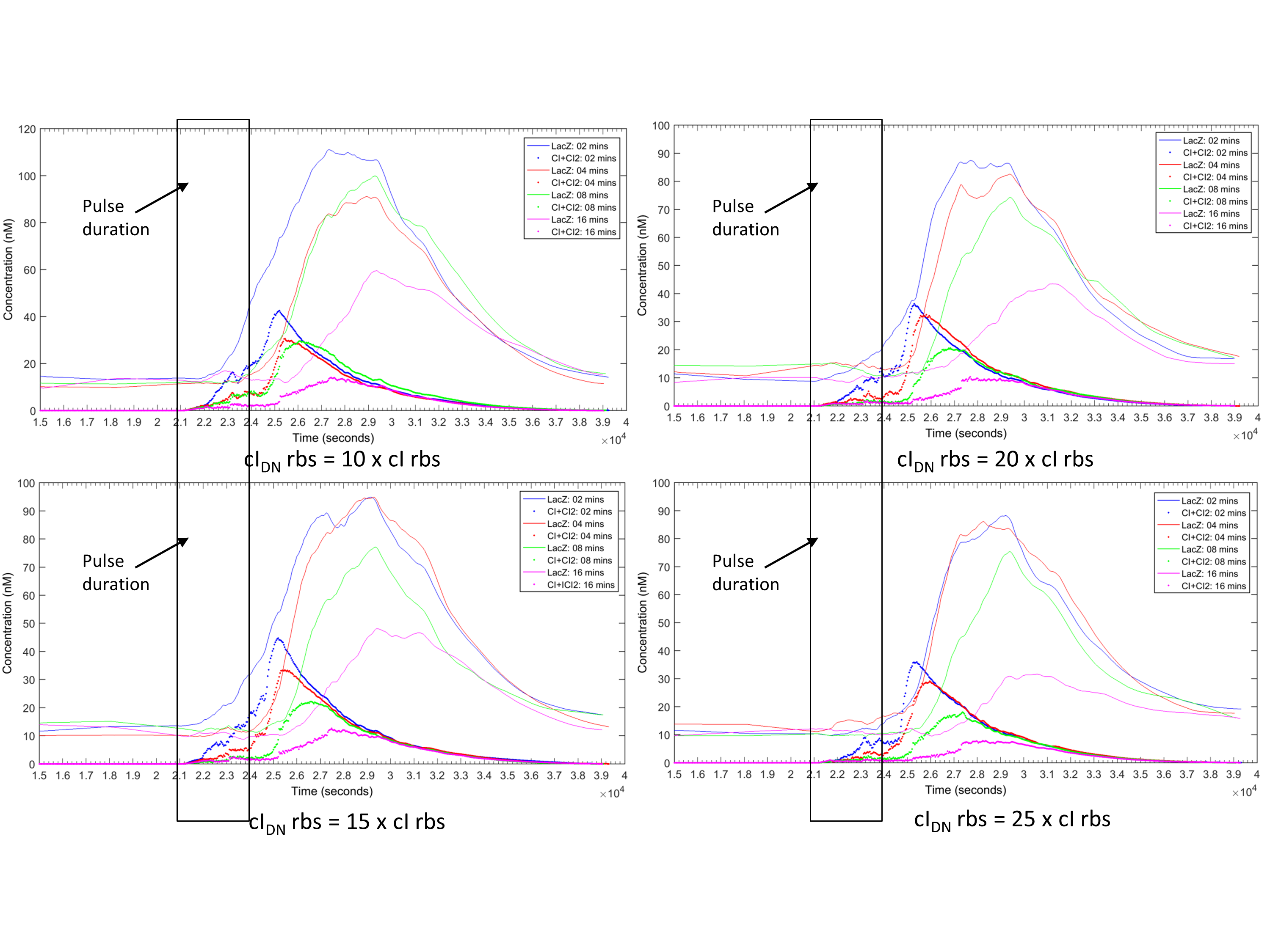

Supplement: S5 Fig — The duration of the pulse, activated at 10.2 CC (20808 sec) and deactivated at 11.7 CC (23868 sec), is 1½ bacterial cell-cycle. The relative strength of RBS1 and RBS2 was varied from 10, 15, 20 and 25. For each RBS1:RBS2 ratio degradation tags with half-life 2, 4, 8 and 16 minutes were used. The response is average of 20 simulation runs. (TIF) [file pone.0167162.s005.tif]

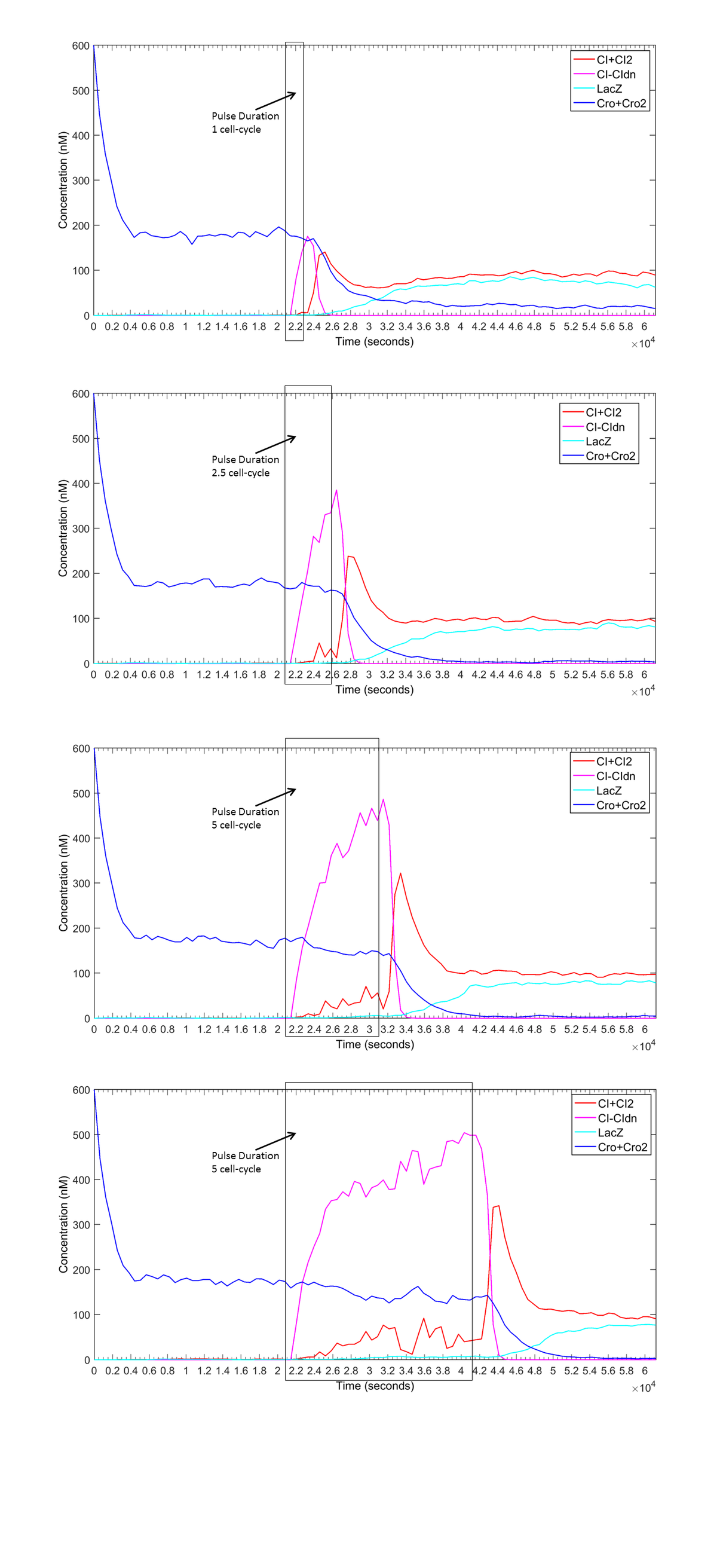

Supplement: S6 Fig — Each circuit was simulation for 30 bacterial CC (30X34 minutes).The duration of the pulse (each activated at 10.2 CC (20808 sec)) was varied from 1CC (2040 sec), 2.5 CC (5100 sec), 5 CC (10200 sec) and 10 CC (20400 sec). Each figure shows the average of 20 simulation runs. (TIF) [file pone.0167162.s006.tif]
