# Supplementary material for: Pulse Detecting Genetic Circuit – A New Design Approach
Source: PLoS One. 2016 Dec 1;11(12):e0167162. doi: 10.1371/journal.pone.0167162 (PMC5131961; doi:10.1371/journal.pone.0167162)
Supplement: S1 File — (DOCX) [file pone.0167162.s007.docx]

**Details of the counter circuit:**

In this section, we present the details of the model for the counter circuit. The pulse detecting circuit model, used in different experiments, consists of a subset of these reactions as the pulse detecting circuit does not have the complete lambda memory device in it. However, in all models we used reactions with the same kinetic parameters. The main components of the model, as shown in Figure 5, are the *cI_DN_* and *cI* genes under the control of P_TetA_ promoter with a degradation tag attached to *cI_DN_* gene, the lambda OR promoter region with both P_RM_ and P_R_ promoters. Both *cI* and *lacZ* genes are placed under the control of P_RM_ promoter and the *cro* gene is placed under the control of P_R_ promoter. The whole simulation was performed considering a single copy of the above components in our model. We assume the *E. Coli* host cell is growing rapidly with a cell division time of 34 minutes in a cell volume of 1 μm^3^ and the concentration of free RNAP is 50 nM according to [25].

Induction of P_TetA_ promoter was simulated by allowing the binding of RNAP at the beginning of the pulse and preventing the same at the end of the pulse. Once RNAP bound in the promoter it can go through isomerization and form an open complex from where the transcription of genes can follow. The *cI_DN_* and *cI* genes were assumed to be separated by 50bp. In our model for each promoter, we considered binding of RNAP and binding site specific binding of different molecules, transcription of genes into mRNAs, translations of genes into proteins, dimerization of proteins, degradation of mRNAs and proteins. Most of the parameters for reactions related to P_RM_ and P_R_ promoters and *cI* and *cro* genes are adopted from the model of -lambda switch by Morelli et al. [25]. Parameters for P_TetA_ promoter are taken from [26]. The notations used in [25] were adopted in representing the molecules and reactions. The notation O(XYZ) refers to the three operators in the O_R_ region, where X, Y and Z are bound to the sites O_R3_, O_R2_, and O_R1_ respectively. X,Y and Z can take a value from {0, R, C, Rp} where 0 indicates an empty site and R, C and Rp indicate a CI dimer, a Cro dimer and an RNA polymerase molecule bound in these operators. Molecules starting with ‘M’ represent mRNAs, e.g. MCI indicates mRNA of *cI*. PtetA represents *TetA* promoter, Rp.PtetA represents the RNA polymerase bound to *TetA* promoter and Rp.PtetAoc represents the open complex of RNA polymerase bound to *TetA* promoter. CI.CIdn represents a *cI_DN_*-*cI* dimer.

The complete listing of the reaction set with kinetic parameters is as follows:

Molecules for the counter circuit

1. CI
2. Cro
3. CI_2_
4. Cro_2_
5. Rp
6. O(000)
7. O(R00)
8. O(0R0)
9. O(00R)
10. O(C00)
11. O(0C0)
12. O(00C)
13. O(Rp00)
14. O(0Rp)
15. O(RR0)
16. O(ROR)
17. O(RC0)
18. O(R0C)
19. O(RRp)
20. O(0RR)
21. O(CR0)
22. O(0RC)
23. O(RpR0)
24. O(C0R)
25. O(0CR)
26. O(Rp0R)
27. O(CC0)
28. O(C0C)
29. O(CRp)
30. O(0CC)
31. O(RpC0)
32. O(Rp0C)
33. O(RRR)
34. O(RRC)
35. O(RCR)
36. O(CRR)
37. O(RpRR)
38. O(RCC)
39. O(CRC)
40. O(CCR)
41. O(RpCR)
42. O(RpRC)
43. O(CCC)
44. O(RpCC)
45. O(RpRp)
46. MCI
47. MCro
48. MLacZ
49. LacZ
50. DLacZ
51. DLacZ1
52. PtetA
53. Rp.PtetA
54. Rp.PtetAoc
55. MCIdn
56. DCI
57. CIdn
58. CI.CIdn

Set of reactions for the counter circuit

1. CI + CI → CI_2_ k=0.628319
2. CI_2_ → CI + CI k=5.705413
3. Cro + Cro → Cro_2_ k=0.628319
4. Cro_2_ → Cro + Cro k=280.199086
5. O(000) + CI_2_ → O(R00) k=0.314159
6. O(R00) → O(000) + CI_2_ k=38.256936
7. O(000) + CI_2_ → O(0R0) k=0.314159
8. O(0R0) → O(000) + CI_2_ k=7.551827
9. O(000) + CI_2_ → O(00R) k=0.314159
10. O(00R) → O(000) + CI_2_ k=0.294263
11. O(000) + Cro_2_ → O(C00) k=0.314159
12. O(C00) → O(000) + Cro_2_ k=0.068319
13. O(000) + Cro_2_ → O(0C0) k=0.314159
14. O(0C0) → O(000) + Cro_2_ k=4.641460
15. O(000) + Cro_2_ → O(00C) k=0.314159
16. O(00C) → O(000) + Cro_2_ k=0.662315
17. O(000) + Rp → O(Rp00) k=0.314159
18. O(Rp00) → O(000) + Rp k=1.490712
19. O(000) + Rp → O(0Rp) k=0.314159
20. O(0Rp) → O(000) + Rp k=0.294263
21. O(R00) + CI_2_ → O(RR0) k=0.314159
22. O(RR0) → O(R00) + CI_2_ k=0.068319
23. O(R00) + CI_2_ → O(ROR) k=0.314159
24. O(ROR) → O(R00) + CI_2_ k=0.294263
25. O(R00) + Cro_2_ → O(RC0) k=0.314159
26. O(RC0) → O(R00) + Cro_2_ k=4.641460
27. O(R00) + Cro_2_ → O(R0C) k=0.314159
28. O(R0C) → O(R00) + Cro_2_ k=0.662315
29. O(R00) + Rp → O(RRp) k=0.314159
30. O(RRp) → O(R00) + Rp k=0.294263
31. O(0R0) + CI_2_ → O(RR0) k=0.314159
32. O(RR0) → O(0R0) + CI_2_ k=0.346100
33. O(0R0) + CI_2_ → O(0RR) k=0.314159
34. O(0RR) → O(0R0) + CI_2_ k=0.003683
35. O(0R0) + Cro_2_ → O(CR0) k=0.314159
36. O(CR0) → O(0R0) + Cro_2_ k=0.068319
37. O(0R0) + Cro_2_ → O(0RC) k=0.314159
38. O(0RC) → O(0R0) + Cro_2_ k=0.662315
39. O(0R0) + Rp → O(RpR0) k=0.314159
40. O(RpR0) → O(0R0) + Rp k=1.490712
41. O(00R) + CI_2_ → O(ROR) k=0.314159
42. O(ROR) → O(00R) + CI_2_ k=38.256936
43. O(00R) + CI_2_ → O(0RR) k=0.314159
44. O(0RR) → O(00R) + CI_2_ k=0.094509
45. O(00R) + Cro_2_ → O(C0R) k=0.314159
46. O(C0R) → O(00R) + Cro_2_ k=0.068319
47. O(00R) + Cro_2_ → O(0CR) k=0.314159
48. O(0CR) → O(00R) + Cro_2_ k=4.641460
49. O(00R) + Rp → O(Rp0R) k=0.314159
50. O(Rp0R) → O(00R) + Rp k=1.490712
51. O(C00) + CI_2_ → O(CR0) k=0.314159
52. O(CR0) → O(C00) + CI_2_ k=7.551827
53. O(C00) + CI_2_ → O(C0R) k=0.314159
54. O(C0R) → O(C00) + CI_2_ k=0.294263
55. O(C00) + Cro_2_ → O(CC0) k=0.314159
56. O(CC0) → O(C00) + Cro_2_ k=1.753314
57. O(C00) + Cro_2_ → O(C0C) k=0.314159
58. O(C0C) → O(C00) + Cro_2_ k=0.662315
59. O(C00) + Rp → O(CRp) k=0.314159
60. O(CRp) → O(C00) + Rp k=0.294263
61. O(0C0) + CI_2_ → O(RC0) k=0.314159
62. O(RC0) → O(0C0) + CI_2_ k=38.256936
63. O(0C0) + CI_2_ → O(0CR) k=0.314159
64. O(0CR) → O(0C0) + CI_2_ k=0.294263
65. O(0C0) + Cro_2_ → O(CC0) k=0.314159
66. O(CC0) → O(0C0) + Cro_2_ k=0.025808
67. O(0C0) + Cro_2_ → O(0CC) k=0.314159
68. O(0CC) → O(0C0) + Cro_2_ k=0.130739
69. O(0C0) + Rp → O(RpC0) k=0.314159
70. O(RpC0) → O(0C0) + Rp k=1.490712
71. O(00C) + CI_2_ → O(R0C) k=0.314159
72. O(R0C) → O(00C) + CI_2_ k=38.256936
73. O(00C) + CI_2_ → O(0RC) k=0.314159
74. O(0RC) → O(00C) + CI_2_ k=7.551827
75. O(00C) + Cro_2_ → O(C0C) k=0.314159
76. O(C0C) → O(00C) + Cro_2_ k=0.068319
77. O(00C) + Cro_2_ → O(0CC) k=0.314159
78. O(0CC) → O(00C) + Cro_2_ k=0.916213
79. O(00C) + Rp → O(Rp0C) k=0.314159
80. O(Rp0C) → O(00C) + Rp k=1.490712
81. O(Rp00) + CI_2_ → O(RpR0) k=0.314159
82. O(RpR0) → O(Rp00) + CI_2_ k=7.551827
83. O(Rp00) + CI_2_ → O(Rp0R) k=0.314159
84. O(Rp0R) → O(Rp00) + CI_2_ k=0.294263
85. O(Rp00) + Cro_2_ → O(RpC0) k=0.314159
86. O(RpC0) → O(Rp00) + Cro_2_ k=4.641460
87. O(Rp00) + Cro_2_ → O(Rp0C) k=0.314159
88. O(Rp0C) → O(Rp00) + Cro_2_ k=0.662315
89. O(Rp00) + Rp → O(RpRp) k=0.314159
90. O(RpRp) → O(Rp00) + Rp k=0.294263
91. O(0Rp) + CI_2_ → O(RRp) k=0.314159
92. O(RRp) → O(0Rp) + CI_2_ k=38.256936
93. O(0Rp) + Cro_2_ → O(CRp) k=0.314159
94. O(CRp) → O(0Rp) + Cro_2_ k=0.068319
95. O(0Rp) + Rp → O(RpRp) k=0.314159
96. O(RpRp) → O(0Rp) + Rp k=1.490712
97. O(RR0) + CI_2_ → O(RRR) k=0.314159
98. O(RRR) → O(RR0) + CI_2_ k=0.407068
99. O(RR0) + Cro_2_ → O(RRC) k=0.314159
100. O(RRC) → O(RR0) + Cro_2_ k=0.662315
101. O(ROR) + CI_2_ → O(RRR) k=0.314159
102. O(RRR) → O(ROR) + CI_2_ k=0.094509
103. O(ROR) + Cro_2_ → O(RCR) k=0.314159
104. O(RCR) → O(ROR) + Cro_2_ k=4.641460
105. O(0RR) + CI_2_ → O(RRR) k=0.314159
106. O(RRR) → O(0RR) + CI_2_ k=38.256936
107. O(0RR) + Cro_2_ → O(CRR) k=0.314159
108. O(CRR) → O(0RR) + Cro_2_ k=0.068319
109. O(0RR) + Rp → O(RpRR) k=0.314159
110. O(RpRR) → O(0RR) + Rp k=1.490712
111. O(RC0) + CI_2_ → O(RCR) k=0.314159
112. O(RCR) → O(RC0) + CI_2_ k=0.294263
113. O(RC0) + Cro_2_ → O(RCC) k=0.314159
114. O(RCC) → O(RC0) + Cro_2_ k=0.130739
115. O(R0C) + CI_2_ → O(RRC) k=0.314159
116. O(RRC) → O(R0C) + CI_2_ k=0.068319
117. O(R0C) + Cro_2_ → O(RCC) k=0.314159
118. O(RCC) → O(R0C) + Cro_2_ k=1.753314
119. O(CR0) + CI_2_ → O(CRR) k=0.314159
120. O(CRR) → O(CR0) + CI_2_ k=0.003683
121. O(CR0) + Cro_2_ → O(CRC) k=0.314159
122. O(CRC) → O(CR0) + Cro_2_ k=0.662315
123. O(C0R) + CI_2_ → O(CRR) k=0.314159
124. O(CRR) →O(C0R) + CI_2_ k=0.094509
125. O(C0R) + Cro_2_ → O(CCR) k=0.314159
126. O(CCR) → O(C0R) + Cro_2_ k=1.753314
127. O(0CR) + CI_2_ → O(RCR) k=0.314159
128. O(RCR) → O(0CR) + CI_2_ k=38.256936
129. O(0CR) + Cro_2_ → O(CCR) k=0.314159
130. O(CCR) → O(0CR) + Cro_2_ k=0.025808
131. O(0CR) + Rp → O(RpCR) k=0.314159
132. O(RpCR) → O(0CR) + Rp k=1.490712
133. O(0RC) + CI_2_ → O(RRC) k=0.314159
134. O(RRC) → O(0RC) + CI_2_ k=0.346100
135. O(0RC) + Cro_2_ → O(CRC) k=0.314159
136. O(CRC) → O(0RC) + Cro_2_ k=0.068319
137. O(0RC) + Rp → O(RpRC) k=0.314159
138. O(RpRC) → O(0RC) + Rp k=1.490712
139. O(CC0) + CI_2_ → O(CCR) k=0.314159
140. O(CCR) → O(CC0) + CI_2_ k=0.294263
141. O(CC0) + Cro_2_ → O(CCC) k=0.314159
142. O(CCC) → O(CC0) + Cro_2_ k=0.407068
143. O(C0C) + CI_2_ → O(CRC) k=0.314159
144. O(CRC) → O(C0C) + CI_2_ k=7.551827
145. O(C0C) + Cro_2_ → O(CCC) k=0.314159
146. O(CCC) → O(C0C) + Cro_2_ k=1.077612
147. O(0CC) + CI_2_ → O(RCC) k=0.314159
148. O(RCC) → O(0CC) + CI_2_ k=38.256936
149. O(0CC) + Cro_2_ → O(CCC) k=0.314159
150. O(CCC) → O(0CC) + Cro_2_ k=0.080354
151. O(0CC) + Rp → O(RpCC) k=0.314159
152. O(RpCC) → O(0CC) + Rp k=1.490712
153. O(RpR0) + CI_2_ → O(RpRR) k=0.314159
154. O(RpRR) → O(RpR0) + CI_2_ k=0.003683
155. O(RpR0) + Cro_2_ → O(RpRC) k=0.314159
156. O(RpRC) → O(RpR0) + Cro_2_ k=0.662315
157. O(Rp0R) + CI_2_ → O(RpRR) k=0.314159
158. O(RpRR) → O(Rp0R) + CI_2_ k=0.094509
159. O(Rp0R) + Cro_2_ → O(RpCR) k=0.314159
160. O(RpCR) → O(Rp0R) + Cro_2_ k=4.641460
161. O(RpC0) + CI_2_ → O(RpCR) k=0.314159
162. O(RpCR) → O(RpC0) + CI_2_ k=0.294263
163. O(RpC0) + Cro_2_ → O(RpCC) k=0.314159
164. O(RpCC) → O(RpC0) + Cro_2_ k=0.130739
165. O(Rp0C) + CI_2_ → O(RpRC) k=0.314159
166. O(RpRC) → O(Rp0C) + CI_2_ k=7.551827
167. O(Rp0C) + Cro_2_ → O(RpCC) k=0.314159
168. O(RpCC) → O(Rp0C) + Cro_2_ k=1.753314
169. O(Rp00) → O(000) + MCI + DLacZ k=0.001000
170. O(RpR0) → O(0R0) + MCI + DLacZ k=0.011000
171. O(Rp0R) → O(00R) + MCI + DLacZ k=0.001000
172. O(RpC0) → O(0C0) + MCI + DLacZ k=0.001000
173. O(Rp0C) → O(00C) + MCI + DLacZ k=0.001000
174. O(RpRR) → O(0RR) + MCI + DLacZ k=0.011000
175. O(RpRC) → O(0RC) + MCI + DLacZ k=0.011000
176. O(RpCR) → O(0CR) + MCI + DLacZ k=0.001000
177. O(RpCC) → O(0CC) + MCI + DLacZ k=0.001000
178. O(RpRp) → O(0Rp) + MCI + DLacZ k=0.001000
179. O(0Rp) → O(000) + Rp + MCro k=0.014000
180. O(RRp) → O(R00) + Rp + MCro k=0.014000
181. O(CRp) → O(C00) + Rp + MCro k=0.014000
182. O(RpRp) → O(Rp00) + Rp + MCro k=0.014000
183. MCI → MCI + CI k=0.034656
184. MCro → MCro + Cro k=0.115520
185. MCro → () k=0.005576
186. DLacZ → Rp k=5
187. DLacZ → DLacZ1 k=45
188. DLacZ1 → Rp + MLacZ k=0.01626
189. MLacZ → MLacZ + LacZ k=0.034656
190. MLacZ → () k=0.005776
191. MCI → () k=0.005776
192. LacZ → () k=0.0000642
193. PtetA + Rp → Rp.PtetA k=0.0142786
194. Rp.PtetA → PtetA + Rp k=0.10
195. Rp.PtetA → Rp.PtetAoc k=0.007
196. Rp.PtetAoc → MCIdn + DCI + PtetA k=0.06494
197. DCI → MCI + Rp k=0.06944
198. MCIdn → () k=0.005776
199. MCIdn → MCIdn + CIdn k=0.005776
200. CI + CIdn → CI.CIdn k=0.628319
201. CI.CIdn → CI + CIdn k=5.705413
202. CIdn → () k=0.002888
203. CI.CIdn → () k=0.002888
204. CI → () k=1.92e-5
205. CI_2_ → () k=1.92e-5
206. Cro → () k=3.85082e-5
207. Cro_2_ → () k=3.85082e-5
